# Relationship between mismatch repair immunophenotype and long-term survival in patients with resected periampullary adenocarcinoma

**DOI:** 10.1186/s12967-018-1444-4

**Published:** 2018-03-14

**Authors:** Margareta Heby, Sebastian Lundgren, Björn Nodin, Jacob Elebro, Jakob Eberhard, Karin Jirström

**Affiliations:** Department of Clinical Sciences Lund, Division of Oncology and Pathology, Lund University, Skåne University Hospital, 221 85 Lund, Sweden

**Keywords:** MMR, Periampullary adenocarcinoma, TMA, Immunohistochemistry, Adjuvant therapy

## Abstract

**Background:**

Periampullary adenocarcinomas, including pancreatic cancer, are a heterogeneous group of tumors with poor prognosis, where classification into intestinal type (I-type) or pancreatobiliary type (PB-type) is a relevant prognostic factor. The clinical significance of deficient mismatch repair (dMMR) in periampullary adenocarcinoma is comparatively unexplored. Herein, we examined the associations of MMR immunophenotype with long-term survival in patients with resected periampullary adenocarcinoma, with particular reference to morphology and adjuvant treatment response.

**Methods:**

MMR protein expression was assessed by immunohistochemistry on tissue microarrays with primary tumors from a retrospective cohort of 175 patients with periampullary adenocarcinoma treated with pancreaticoduodenectomy during 2001–2011 in Malmö and Lund University Hospitals, Sweden. Cox proportional hazards models were applied to calculate hazard ratios (HR) and 95% confidence intervals (CI).

**Results:**

After a mean follow-up of 46.5 (1.9–185.1) months, 35 patients (20.3%) were alive, 24 with I-type and 11 with PB-type tumors. MMR protein expression could be evaluated in 172 cases, in which dMMR was denoted in 20 (11.6%) cases, 13/63 (20.6%) in I-type and 7/109 (6.4%) in PB-type tumors. dMMR was associated with a significantly prolonged overall survival in the entire cohort (HR = 0.28, 95% CI 0.13–0.57), and in I-type tumors (HR = 0.20, 95% CI 0.06–0.68), however not independent of conventional prognostic factors. In PB-type tumors, dMMR was not prognostic, but there was a significant negative interaction between dMMR and adjuvant treatment (p_interaction_ = 0.015).

**Conclusions:**

dMMR is more frequent in I-type compared to PB-type periampullary adenocarcinoma, and is a prognostic factor for long-term survival only in the former. The finding of the small number of PB-type tumors with dMMR potentially lacking benefit from adjuvant chemotherapy is however noteworthy and merits further validation.

**Electronic supplementary material:**

The online version of this article (10.1186/s12967-018-1444-4) contains supplementary material, which is available to authorized users.

## Background

The periampullary region describes the anatomical location around the ampulla of Vater. Adenocarcinoma of this region includes tumors originating in the distal bile duct, pancreas, ampulla of Vater and the periampullary duodenum. These tumors are a heterogeneous group of neoplasms, with pancreatic cancer being the most common type. The periampullary adenocarcinomas are divided into two different morphological types, i.e. pancreatobiliary type (PB-type), and intestinal type (I-type). PB-type tumors, which include pancreatic cancer, distal bile duct cancer, and some of the ampullary carcinomas, have a worse prognosis and are associated with significantly shorter survival rates compared to I-type tumors [1, 2], which include duodenal carcinoma and some of the ampullary carcinomas. Hence, tumor morphology provides important prognostic information. The overall 5-year survival is 7% for all stages combined and the median survival is approximately 6 months [3]. In R0-resected pancreatic cancer the 5-year survival is 20% with a median survival of 24 months after resection, whereas for ampullary adenocarcinoma the prognosis is somewhat better with a median survival of 36–44 months after resection [4–6]. Tumors in this region are mainly diagnosed at a late stage, with only 15–20% being resectable at presentation [7], resectability often being limited by early local invasion of the surrounding anatomical structures such as arterial vessels or distant metastasis. The only cure is surgery with R0 resection, and for borderline resectable tumors neoadjuvant chemotherapy should be offered since approximately one-third of these tumors can be converted to resectability [8]. Randomized trials in the 1990s showed that adjuvant chemotherapy for pancreatic cancer prolongs life, as compared to observation [9, 10]. Several studies have confirmed these results and adjuvant treatment has now become standard of care. In the palliative setting, comprising almost 80% of the cases, treatment consists of different chemotherapy combinations and/or radiotherapy, however the effect is often minimal and short-lived. Therefore, there is a great need for additional molecular-based biomarkers, to better define clinically relevant subgroups of these tumors, so as to enable improved personalized treatment strategies. Immune-modulating therapy, e.g. targeting the programmed death receptor 1 (PD-1) pathway, is a treatment option that has shown promising results in various types of tumors, but the efficacy in periampullary cancer remains unclear and checkpoint inhibition in pancreatic cancer has been disappointing thus far [11, 12]. Mismatch repair (MMR) immunophenotype, is a putative biomarker of response to such therapies. Epigenetic or mutational inactivation of certain MMR genes, including MutL homolog 1 (MLH1), post-meiotic segregation 2 (PMS2), MutS protein homolog 2 (MSH2) and MutS protein homolog 6 (MSH6), typically results in microsatellite instability (MSI) which means a failure to repair errors that occur during replication of repetitive DNA sequences [13]. Consequently, these tumors contain thousands of mutations that may produce neoantigens that can be recognized and targeted by T cells, changes that have been linked to increased sensitivity to checkpoint inhibitors, e g programmed death receptor 1 (PD-1) blockade [14]. MMR deficient tumors have been demonstrated to respond to treatment with the anti-PD-1 immune checkpoint inhibitor pembrolizumab [15]. In colorectal cancer (CRC), high levels of MSI have been found to predict a better overall prognosis and an increased benefit from immune based therapies [16], however there are reports suggesting that in stage IV disease, the prognosis of CRC with high MSI is poorer than for microsatellite-stable cases [17]. The prevalence of dMMR and high MSI in pancreatic cancer has in some studies been reported to range between 13 and 22% in primarily surgically resected patients [18, 19], but still remains poorly characterized. Some recently published studies conclude that MSI positivity in resectable pancreatic cancer may be a favorable prognostic factor [18, 20], and one study on ampulla of Vater adenocarcinoma demonstrated that patients with MSI high tumors had a significantly longer overall survival [21]. In conclusion, the role of MMR immunophenotype as a potential prognostic and predictive biomarker for response to adjuvant and immunotherapy in periampullary adenocarcinoma remains unclear, hence further investigation is warranted. The aim of this study was therefore to examine the frequency of MMR deficiency in a retrospective cohort of periampullary adenocarcinoma, with particular reference to tumor morphology and relationship with long-term survival and adjuvant treatment response.

## Methods

### Patients

The study cohort consists of a previously described retrospective consecutive series of 175 patients with primary periampullary adenocarcinomas [22–26]. All patients were subjected to pancreaticoduodenectomy at the University hospitals of Lund and Malmö, Sweden, from January 1 2001 until December 31 2011. In the full cohort of 175 cases the anatomical origin was 14 duodenal, 70 ampullary, 45 distal bile duct and 46 pancreatic, in all 110 PB-type and 65 I-type adenocarcinomas. Data on survival were gathered from the Swedish National Civil Register. Follow-up started at the date of surgery and ended at death, or at March 31 2017, whichever came first. Clinical data regarding adjuvant treatment, recurrence and clinicopathological data were obtained retrospectively from medical records and the last update reaches until March 31 2017. The patients were identified through broad searches in the pathology database, and all haematoxylin and eosin stained slides were re-evaluated by one pathologist (JEL), blinded to the original report and outcome, with the decision on tumor origin and morphological type being based on several criteria, as previously described [22].

The study was approved by the Ethics Committee of Lund University (ref no. 445/07).

### Tissue microarray construction

Tissue microarrays (TMAs) were constructed using a semi-automated arraying device (TMArrayer, Pathology Devices, Westminister, MD, USA). A standard set of three tissue cores (1 mm) were obtained from each of the 175 primary tumors and from lymph node metastases from 105 of the cases, whereby one to three lymph node metastases were sampled in each case. In addition, adjacent benign-appearing pancreatic tissue was sampled from 50 cases using two (1 mm) tissue cores.

### Immunohistochemistry and staining evaluation

For immunohistochemical analysis of MMR proteins, 4 μm TMA sections were automatically pretreated in the PT-link system (Dako, Glostrup, Denmark) with heat induced epitope retrieval (HIER) in TRS pH 9 (Dako, catalogue nr K8004), 20 min in 97 °C, and then stained in an automated immunostainer (Autostainer Link 48, Dako) using the Dako EnVision™FLEX+ Detection System, Peroxidase/DAB, Rabbit/Mouse (catalogue nr K8002), with the following ready- to -use monoclonal antibodies: MLH1 (clone ES05, part nr IR07961-2, Dako), PMS2 (clone EP51, part nr IR8761-2, Dako), MSH2 (clone FE11, part nr IR08561-2, Dako) and MSH6 (clone EP49, part nr IR08661-2, Dako). After IHC staining, slides were rinsed 5 min in tap water, dehydrated and mounted with Tissue Tek Prisma (Sakura Finetek, Alphen aan den Rijn, Netherlands).

Staining of MMR was evaluated by three independent observers (MH, SL and KJ), who were blinded to clinical and outcome data. Immunohistochemical stainings were denoted as negative when all tumor cells showed loss of nuclear staining. Surrounding stromal cells and tumor infiltrating lymphocytes served as internal controls for each TMA core. Cases lacking positive internal controls were excluded. Deficient MMR (dMMR) was defined as negative staining for MLH1, PMS2, MSH2 or MSH6, and proficient MMR (pMMR) was defined as positive staining for all four MMR proteins. Immunohistochemical analysis of CD3^+^ lymphocytes, CD56^+^ natural killer (NK)/NKT cells and CD68^+^ and CD163^+^ macrophages had been performed previously [27, 28]. For immunohistochemical analysis of FoxP3 and CD8^+^ T cells, 4 μm TMA-sections were automatically pre-treated using the PT Link system and then stained in an Autostainer Plus (Dako; Glostrup, Denmark) with the anti-FoxP3 antibody (clone 236A/E7, mouse, dilution 1:200, Abcam, Cambridge, UK), and the anti-CD8 antibody (clone C8/144B, mouse; dilution, 1:50; product M7103; Dako). The total number (intratumoral, tumor-adjacent and stromal) CD8^+^ immune cells in each core was calculated by automated analysis using the co-localization algorithm within the Halo image analysis software (Indica Labs, Corrales, NM, USA). The number of FoxP3^+^ cells (intratumoral, tumor-adjacent and stromal) was calculated manually. A median value of the cores was calculated and used in the analyses.

### Statistical analysis

Chi square test was applied to analyze the relationship between MMR immunophenotype and categorical clinicopathological parameters, whereas Mann–Whitney U test was used in continuous variables such as age, tumor size and immune cells. Three patients were excluded from the survival analyses; two with I-type adenocarcinomas who died within 1 month from surgery due to complications and one with PB-type adenocarcinoma who emigrated 5 months after surgery. Kaplan–Meier analysis and log rank test were applied to estimate differences in long-term overall survival (OS), in strata according to dMMR/pMMR. Hazard ratios (HR) for death and recurrence were calculated by Cox regression proportional hazard’s modeling in unadjusted analysis and in a multivariable model. Only factors with a *p* value < 0.05 were included in the multivariable analysis. To estimate the interaction effect between adjuvant treatment and MMR immunophenotype, the following interaction variable was constructed; any adjuvant treatment (±) × MSI (±). The proportional hazard (PH) assumption was tested using Cox regression with a time-dependent covariate analysis, whereby the PH assumption was considered to be satisfied when the factor × time interaction was non-significant. The PH assumption was also evaluated graphically using log-minus-log plots. All tests were two sided. p-values < 0.05 were considered significant. All statistical analyses were performed using IBM SPSS Statistics version 22.0 (SPSS Inc., Chicago, IL, USA).

## Results

Three patients were excluded from the survival analyses; two with I-type adenocarcinomas who died within 1 month from surgery due to complications and one with PB-type adenocarcinoma who emigrated 5 months after surgery. Mean and median follow-up time was 46.5 and 29.7 months (Q1 16.6 and Q3 65.6 months) respectively (range 1.9–185.1 months), and in March 2017, 35 patients (20.3%) were alive, 24 with I-type and 11 with PB-type tumors. Recurrent disease was denoted in 124 patients (72.1%), 31 with I-type and 93 with PB-type tumors. Thus, 50.8% of patients with I-type tumors did not have recurrence at follow-up in March 2017. Two of the 24 patients alive with I-type tumor morphology had recurrent disease and were of pMMR immunophenotype. Ten patients with I-type and 5 patients with PB-type tumors died without signs of recurrence and thus from other causes. Adjuvant chemotherapy was given in 77 (44.8%) cases, 18/63 (28.6%) I-type and 59/109 (54.1%) PB-type tumors. Of patients with I-type tumors, 50% received 5-fluorouracil (5-FU)-based chemotherapy, and 50% gemcitabine-based chemotherapy, single or in combination. Corresponding numbers in patients with PB-type tumors were less than 20% 5-FU-based and the rest gemcitabine-based treatment.

### MMR immunophenotype

**Fig. 1:**
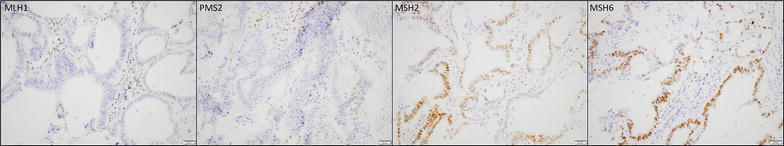
Sample immunohistochemical images of MMR protein expression in an MMR deficient duodenal cancer (MHL1 and PMS2 negative, MSH2 and MSH6 positive)

**Fig. 2:**
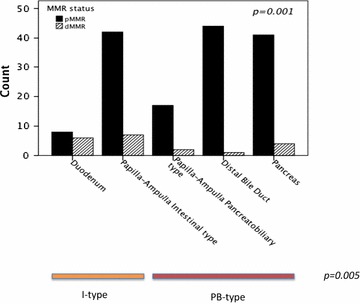
The distribution of MMR immunophenotype according to anatomical subsite

Sample immunohistochemical images of MMR protein expression are shown in Fig. 1. MMR protein expression could be evaluated in 172 cases, in which dMMR was denoted in 20 (11.6%) cases, 13/63 (20.6%) in I-type and 7/109 (6.4%) in PB-type tumors. The distribution of loss of different MMR proteins in relation to morphological type is shown in Table 1. Loss of MSH6 was all over the most common, and seen in 71.4% of PB-type tumors. In I-type tumors the distribution of different combinations was more even. The distribution of MMR immunophenotype according to anatomical subsite is presented in Fig. 2. In tumors with duodenal origin, 43% were denoted as having dMMR. There was no discordance in MMR immunophenotype between primary tumors and lymph node metastases. In the two cases that had received neoadjuvant chemotherapy, both tumors were pMMR and thus included in the analyses, since neoadjuvant treatment had not affected the expression of any MMR protein.

**Table 1:** The distribution of loss of different MMR proteins in relation to morphological type

n (%)	MLH1	PMS2	MSH2	MSH6
All				
1 (5.0)	Intact	Loss	Intact	Uninterpretable^a^
1 (5.0)	Loss	Loss	Intact	Loss
3 (15.0)	Loss	Loss	Intact	Intact
4 (20.0)	Intact	Loss	Intact	Intact
3 (15.0)	Intact	Intact	Loss	Loss
8 (40.0)	Intact	Intact	Intact	Loss
I-type				
1 (7.7)	Loss	Loss	Intact	Loss
3 (23.1)	Loss	Loss	Intact	Intact
4 (30.1)	Intact	Loss	Intact	Intact
2 (15.4)	Intact	Intact	Loss	Loss
3 (23.1)	Intact	Intact	Intact	Loss
PB-type				
1 (14.3)	Intact	Loss	Intact	Uninterpretable^a^
1 (14.3)	Intact	Intact	Loss	Loss
5 (71.4)	Intact	Intact	Intact	Loss

^a^Lack of positive internal control staining in lymphocytes or stromal cells

### Associations of MMR immunophenotype with clinicopathological factors and tumor-infiltrating immune cells

**Fig. 3:**
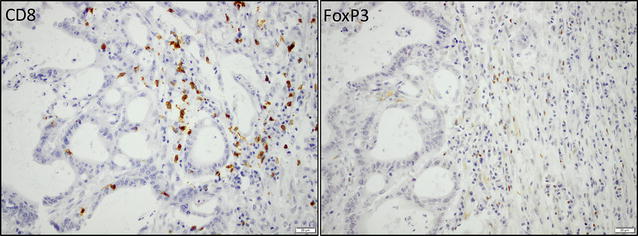
Sample immunohistochemical images of CD8+ (membranous/cytoplasmic staining) and FoxP3 (nuclear staining) lymphocytes in the same tumor as in Fig. 1

The associations of MMR immunophenotype with clinicopathological factors and tumor-infiltrating immune cells in the entire cohort, I-type and PB-type tumors, respectively, are shown in Table 2. In the entire cohort, dMMR was significantly associated with I-type morphology (p = 0.003), N0-stage (p = 0.002), perineural growth (p = 0.003), absence of tumor growth in lymphatic vessels (p = < 0.001) and in peripancreatic fat (p = 0.002), infiltration of CD8^+^ T cells (p = 0.035), infiltration of CD56^+^ cells (p = 0.029) and with no adjuvant chemotherapy (p = 0.040). When divided into morphological subtype, dMMR was significantly associated with tumor origin in the duodenum (p = 0.005), larger tumor size (p = 0.006), absence of tumor growth in lymphatic vessels (p = 0.002), and infiltration of CD8^+^ T cells (p = 0.012) in I-type tumors. In PB-type tumors there was a significant association between dMMR and N0-stage (p = 0.002), absence of tumor growth in lymphatic vessels (p = 0.018) and in peripancreatic fat (p = 0.021), and infiltration of CD56^+^ cells (p = 0.046). The density of CD8^+^ T cells and CD56^+^ NK/NKT cells was significantly higher in dMMR tumors, but there were no significant associations between dMMR and the other investigated immune cell subsets. Sample immunohistochemical images of CD8 + and FoxP3 lymphocytes in the same tumor as in Fig. 1 are shown in Fig. 3.

**Table 2:** The association between MMR immunophenotype with clinicopathological factors in I-type tumors, PB-type tumors, and the entire cohort respectively

	Intestinal type			Pancreatobiliary type			All		
	pMMR (n = 50)	dMMR (n = 13)	*p*	pMMR (n = 102)	dMMR (n = 7)	*p*	pMMR (n = 152)	dMMR (n = 20)	*p*
Age									
(Median, range)	66.5 (38.0–79.0)	67.0 (48.0–83.0)	0.905	67.0 (44.0–81.0)	62.0 (58.0–76.0)	0.669	67.0 (38.0–81.0)	65.5 (48.0–83.0)	0.635
Sex									
Women	26 (52.0)	8 (61.5)	0.539	46 (45.1)	4 (57.1)	0.538	72 (47.4)	12 (60.0)	0.289
Men	24 (48.0)	5 (38.5)		56 (54.9)	3 (42.9)		80 (52.6)	8 (40.0)	
Tumor origin									
Duodenum	8 (16.0)	6 (46.2)	0.005				8 (5.3)	6 (28.6)	0.003
Ampulla intestinal type	42 (84.0)	7 (53.8)					42 (27.6)	7 (35.0)	
Ampulla pancreatobiliary type				17 (16.7)	2 (28.6)	0.860	17 (11.2)	2 (10.0)	
Distal bile duct				44 (43.1)	1 (14.3)		44 (28.9)	1 (5.0)	
Pancreas				41 (40.2)	4 (57.1)		41 (27.0)	4 (20.0)	
Tumor size mm									
(Median, range)	25.0 (5.0–60.0)	40.0 (13.0–90.0)	0.006	30.0 (9.0–70.0)	30.0 (5.0–35.0)	0.283	30.0 (5.0–70.0)	33.0 (5.0–90.0)	0.107
Differentiation grade									
Well-moderate	24 (48.0)	7 (53.8)	0.707	37 (36.3)	4 (57.1)	0.272	61 (40.1)	11 (55.0)	0.206
Poor	26 (52.0)	6 (46.2)		65 (63.7)	3 (42.9)		91 (59.9)	9 (45.0)	
T-stage									
T1	4 (8.0)	0	0.736	2 (2.0)	0	0.966	6 (3.9)	0	0.420
T2	9 (18.0)	2 (15.4)		11 (10.8)	1 (14.3)		20 (13.2)	3 (15.0)	
T3	19 (38.0)	6 (46.2)		73 (71.6)	5 (71.4)		92 (60.5)	11 (55.0)	
T4	18 (36.0)	5 (38.5)		16 (15.7)	1 (14.3)		34 (22.4)	6 (30.0)	
N-stage									
N0	24 (48.0)	9 (69.2)	0.348	26 (25.5)	6 (85.7)	0.002	50 (32.9)	15 (75.0)	0.002
N1	17 (34.0)	2 (15.4)		44 (43.1)	1 (14.3)		61 (40.1)	3 (15.0)	
N2	9 (18.0)	2 (15.4)		32 (31.4)	0		41 (27.0)	2 (10.0)	
Margins									
R0	13 (26.0)	4 (30.8)	0.730	6 (5.9)	0	0.511	19 (12.5)	4 (20.0)	0.356
R1–Rx	37 (74.0)	9 (69.2)		96 (94.1)	7 (100.0)		133 (87.5)	16 (80.0)	
Perineural growth									
No	33 (66.0)	11 (84.6)	0.193	21 (20.6)	3 (42.9)	0.171	54 (35.5)	14 (70.0)	0.003
Yes	17 (34.0)	2 (15.4)		81 (79.4)	4 (57.1)		98 (64.5)	6 (30.0)	
Invasion of lymphatic vessels									
No	18 (36.0)	11 (84.6)	0.002	29 (28.4)	5 (71.4)	0.018	47 (30.9)	16 (80.0)	0.000
Yes	32 (64.0)	2 (15.4)		72 (71.6)	2 (28.6)		105 (69.1)	4 (20.0)	
Invasion of blood vessels									
No	45 (90.0)	13 (100.0)	0.235	67 (65.7)	5 (71.4)	0.757	112 (73.7)	18 (90.0)	0.111
Yes	5 (10.0)	0		35 (3437)	2 (28.6)		40 (26.3)	2 (10.0)	
Growth in peripancreatic fat									
No	31 (62.0)	10 (76.9)	0.315	20 (19.6)	4 (57.1)	0.021	51 (33.6)	14 (70.0)	0.002
Yes	19 (38.0)	3 (23.1)		82 (80.4)	3 (42.9)		101 (66.4)	6 (30.0)	
Adjuvant chemotherapy									
None	35 (70.0)	10 (76.9)	0.094	46 (45.1)	4 (57.1)	0.177	81 (53.3)	14 (70.0)	0.040
5FU-analogue	5 (10.0)	0		8 (7.8)	0		13 (8.6)	0 (0.0)	
Gemcitabine	7 (14.0)	0		43 (42.2)	2 (28.6)		50 (32.9)	2 (10.0)	
Gemcitabine + capecitabine	0	1 (7.7)		3 (2.9)	0		3 (2.0)	1 (5.0)	
Oxaliplatin + 5-FU analogue	2 (4.0)	2 (15.4)		1 (1.0)	0		3 (2.0)	2 (10.0)	
Gemcitabine + oxaliplatin	1 (2.0)	0		1 (1.0)	1 (12.5)		2 (1.3)	1 (5.0)	
Immune cells									
CD3^+^	206 (6–795)	246 (59–559)	0.156	129 (22–546)	44 (26–695)	0.092	148.5 (6–795)	227 (26–695)	0.471
*Missing*	0	0		0	0		0	0	
CD8^+^	55 (2–180)	114 (2–180)	0.012	45 (4–175)	37 (1–200)	0.844	47.5 (2–180)	101.5 (1–200)	0.035
*Missing*	1	0		3	0		4	0	
FOXP3^+^	33 (0–110)	40 (1–103)	0.497	26 (1–137)	15 (0–119)	0.420	27.5 (0–137)	38 (0–119)	0.560
*Missing*	0	0		2	1		2	1	
CD68^+^	87 (29–350)	74 (19–182)	0.490	98 (25–230)	97 (36–230)	0.645	93 (25–350)	82 (19–229)	0.455
*Missing*	0	0		1	1		1	1	
CD163^+^	130 (35–250)	120 (44–190)	0.755	140 (49–275)	162 (92–200)	0.241	136 (35–275)	131 (44–200)	0.885
*Missing*	1	0		4	1		5	1	
CD56^+^	1 (0–9)	2 (0–23)	0.370	1 (0–12)	3 (0–33)	0.046	1 (0–12)	2 (0–33)	0.029
*Missing*	0	0		0	0		0	0

### Prognostic and potential predictive value of MMR immunophenotype

**Fig. 4:**
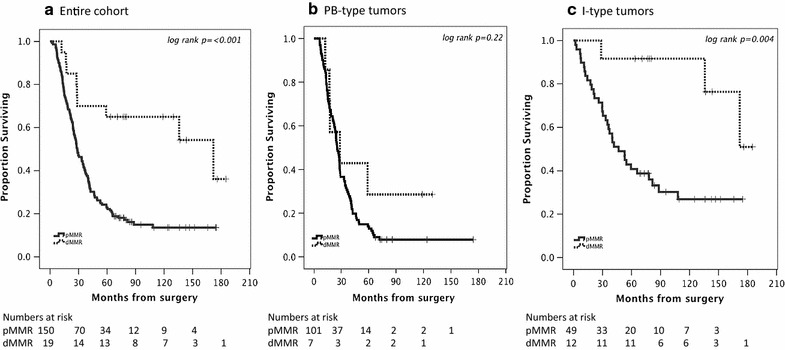
Kaplan–Meier estimates of overall survival in the entire cohort (**a**), in PB-type (**b**) and I-type (**c**) tumors stratified by MMR immunophenotype

**Fig. 5:**
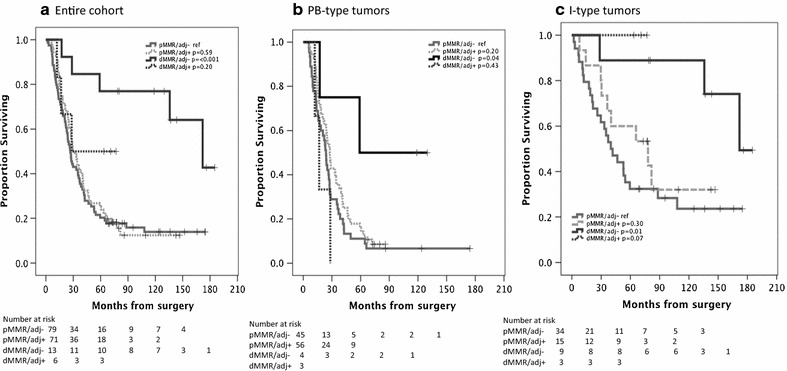
Kaplan–Meier analysis of overall survival in strata according to MMR immunophenotype and adjuvant treatment in the entire cohort (**a**), PB-type (**b**), and I-type tumors (**c**)

As demonstrated in Fig. 4, Kaplan–Meier analysis revealed a significant association between dMMR and prolonged overall survival in the entire cohort (p = <0.001) and in I-type tumors (p = 0.004), but not in PB-type tumors. Cox proportional hazards analyses of OS according to MMR immunophenotype are shown in Table 3. The time-dependent covariate was non-significant for MMR immunophenotype, and therefore, the factor × time interaction term was dropped from the model. The proportional hazard assumption was also considered to be satisfied with graphical evaluation using log-minus-log plots (data not shown). The associations between MMR immunophenotype and OS were confirmed in univariable Cox regression analysis in the entire cohort (HR = 0.28, 95% CI 0.13–0.57) and in I-type tumors (HR = 0.20, 95% CI 0.06–0.68). However, none of these associations were significant when adjusted for conventional prognostic factors. Similar findings were seen for RFS according to MMR immunophenotype, with significant values in the entire cohort and in I-type tumors, but not in PB-type tumors, and not when adjusted for conventional prognostic factors (Additional file 1). Kaplan–Meier analysis of overall survival in strata according to MMR immunophenotype and adjuvant treatment is presented in Fig. 5. In the entire cohort (Fig. 5a) and in PB-type tumors (Fig. 5b), patients with dMMR tumors who had not received adjuvant chemotherapy had the best prognosis, and in I-type tumors, patients with dMMR tumors had the best prognosis irrespective of adjuvant chemotherapy (Fig. 5c). However, and notably, patients with dMMR PB-type tumors who had received adjuvant treatment had the shortest OS (Fig. 5b), and there was a significant negative interaction between dMMR and adjuvant treatment (p_interaction_ = 0.015).

**Table 3:** Unadjusted and adjusted hazard ratios of the impact of MMR immunophenotype on overall survival in I-type tumors, PB-type tumors, and the entire cohort

	Intestinal type			Pancreatobiliary type			All		
	n (events)	Unadjusted	Adjusted	n (events)	Unadjusted	Adjusted	n (events)	Unadjusted	Adjusted
		HR (95% CI)	HR (95% CI)		HR (95% CI)	HR (95% CI)		HR (95% CI)	HR (95% CI)
Age									
Continuous	63 (39)	1.01 (0.98–1.05)	–	109 (98)	0.99 (0.97–1.02)	–	172 (137)	1.01 (0.99–1.03)	–
Gender									
Female	35 (17)	1.00	1.00	51 (45)	1.00	–	86 (62)	1.00	–
	34 (16)*								
Male	28 (22)	*2.01 (1.05–3.84)*	1.60 (0.79–3.24)	58 (53)	1.15 (0.77–1.71)		86 (75)	*1.40 (1.00–1.97)*	
	27 (21)*								
Tumor origin									
Intestinal		–	–		–	–	63 (39)	1.00	1.00
							61 (37)*		
Pancreatobiliary type		–	–		–	–	109 (98)	*2.54 (1.73–3.73)*	*1.61 (1.01–2.55)*
							108 (98)*		
Tumor size									
Continuous	63 (39)	1.00 (0.98–1.02)	–	109 (98)	*1.04 (1.02–1.06)*	*1.02 (1.00–1.04)*	172 (137)	*1.02 (1.00–1.03)*	1.01 (0.99–2.55)
				108 (98)*			169 (135)*		
T-stage									
T1–T2	16 (8)	1.00	–	15 (10)	1.00	1.00	31 (18)	1.00	1.00
				14 (10)*			28 (16)*		
T3–T4	47 (31)	1.72 (0.79–3.74)		94 (88)	*2.47 (1.28–4.77)*	1.06 (0.50–2.27)	141 (119)	*2.34 (1.42–3.86)*	1.21 (0.66–2.21)
				94 (88)*			141 (119)*		
N-stage									
N0	35 (19)	1.00	–	32 (25)	1.00	1.00	67 (44)	1.00	1.00
				31 (25)*			64 (42)*		
N1–2	28 (20)	1.56 (0.83–2.94)		77 (73)	*2.23 (1.41–3.54)*	*2.06 (1.16–3.65)*	105 (93)	*2.19 (1.52–3.16)*	1.34 (0.88–2.05)
				77 (73)*			105 (93)*		
Differentiation grade									
Well-moderate	31 (17)	1.00	–	42 (32)	1.00	1.00	73 (49)	1.00	1.00
				41 (32)*			71 (48)*		
Poor	32 (22)	1.70 (0.90–3.21)		67 (66)	*2.63 (1.70–4.09)*	*2.05 (1.24–3.39)*	99 (88)	*2.24 (1.57–3.18)*	*1.65 (1.09–2.48)*
				67 (66)*			98 (87)*		
Involved margins, status									
R0	18 (5)	1.00	1.00	7 (5)	1.00	–	25 (10)	1.00	1.00
	17 (4)*						23 (9)*		
R1 and Rx	45 (34)	*3.30 (1.29–8.46)*	2.89 (0.94–8.85)	102 (93)	2.42 (0.98–6.00)		147 (127)	*3.57 (1.87–6.83)*	*2.23 (1.10–4.51)*
	44 (33)*						146 (126)*		
Lymphatic growth									
Absent	28 (10)	1.00	1.00	35 (27)	1.00	1.00	63 (37)	1.00	1.00
	28 (10)*			34 (27)*			62 (37)*		
Present	35 (29)	*3.94 (1.90–8.18)*	1.77 (0.77–4.09)	74 (71)	*1.78 (1.13–2.79)*	1.09 (0.65–1.83)	109 (100)	*2.54 (1.73–3.73)*	1.33 (0.85–2.07)
	33 (27)*			74 (71)*			107 (98)*		
Vascular growth									
Absent	58 (34)	1.00	1.00	73 (62)	1.00	1.00	131 (96)	1.00	1.00
	56 (32)*			72 (62)*			128 (94)*		
Present	5 (5)	*6.68 (2.40–18.62)*	*4.69 (1.48–14.84)*	36 (36)	*2.47 (1.61–3.80)*	*2.20 (1.38–3.52)*	41 (41)	*3.49 (2.37–5.15)*	*2.18 (1.43–3.34)*
	5 (5)*			36 (36)*			41 (41)*		
Perineural growth									
Absent	43 (21)	1.00	1.00	25 (19)	1.00	1.00	68 (40)	1.00	1.00
	42 (20)*			24 (19)*			66 (39)*		
Present	20 (18)	*2.81 (1.48–5.35)*	1.36 (0.57–3.21)	84 (79)	*1.96 (1.18–3.25)*	0.88 (0.48–1.61)	104 (97)	*2.96 (2.02–4.43)*	1.06 (0.66–1.71)
	19 (17)*			84 (79)*			103 (96)*		
Growth in peripancreatic fat									
Absent	42 (20)	1.00	1.00	25 (20)	1.00	1.00	67 (40)	1.00	1.00
	40 (18)*			24 (20)*			64 (38)*		
Present	21 (19)	*3.65 (1.86–7.16)*	1.78 (0.73–4.33)	84 (78)	*1.75 (1.06–2.88)*	0.99 (0.56–1.77)	105 (97)	*3.00 (2.03–4.43)*	1.37 (0.85–2.22)
	21 (19)*			84 (78)*			105 (97)*		
Adjuvant treatment									
None	45 (30)	1.00	–	50 (44)	1.00	–	95 (74)	1.00	–
Any	18 (9)	0.69 (0.33–1.47)		59 (54)	0.96 (0.64–1.42)		77 (63)	1.09 (0.78–1–53)	
MMR									
pMMR	49 (34)	1.00	1.00	101 (93)	1.00	1.00	150 (127)	1.00	1.00
	49 (34)*			101 (93)*			150 (127)*		
dMMR	12 (3)	*0.20 (0.06–0.68)*	0.36 (0.09–1.42)	7 (5)	0.57 (0.23–1.42)	1.24 (0.46–3.37)	19 (8)	*0.28 (0.13–0.57)*	0.52 (0.23–1.19)
	12 (3)*			7 (5)*			19 (8)*		

Italic values indicate significance at *p* < 0.05

*Number and events for the adjusted analysis

## Discussion

Despite improvements in the treatment of other solid tumors, e.g. with immune-modulating therapies, overall survival for patients with periampullary adenocarcinoma, continues to be poor. By year 2030, pancreatic adenocarcinoma is predicted to become the second leading cause of cancer death after lung/bronchus cancer [29, 30]. Apart from the prognostic information provided by standard histopathological parameters and CA19-9, no prognostic or predictive biomarkers have yet been introduced into clinical practice. Thus, there is a great need to identify additional biomarkers for improved treatment stratification of patients with periampullary adenocarcinoma, including pancreatic cancer. In this study, we evaluated the prognostic and predictive impact of MMR immunophenotype in periampullary adenocarcinoma with regard to morphological type and adjuvant therapy. In the herein investigated cohort, dMMR was denoted in 11.6% of the cases, which is in line with findings in previous studies [18, 19]. MMR proteins functionally interact in heterodimers, where MLH1 and MSH2 are obligatory proteins in their respective heterodimer and their mutational or epigenetic inactivation leads to destabilization of the corresponding binding partners and results in complete loss of MMR activity [31]. However, other immunohistochemical patterns have been described in pancreatic cancer with loss of MSH2 but not MSH6 [18] and in Lynch syndrome, where the multitude of disease-predisposing mutations may have variable effects on epitope expression, from complete loss to weak or retained expression for one or both heterodimerizing proteins [32, 33]. Since limitations to immunohistochemistry exist, such as the antibodies used in the analysis, the staining pattern, the biopsy sample size and the risk of false negative cases, all results should always be interpreted with this in mind. In our study, a false negative sample (interpreted as dMMR) would alter the results profoundly given the small sample size of tumors with dMMR. However, immunohistochemistry is often the first choice for screening to identify patients for genetic testing, and given its fairly low cost and availability, this method is widely used in the clinical setting as opposed to MSI-testing [34]. The majority of cases with PB-type tumors in this study had lost the expression of MSH6, whereas in I-type tumors, the dominating immunophenotype was loss of PMS2 expression. Our results support the previously described association between dMMR and a prolonged survival in patients with pancreatic cancer [18, 20] and ampulla of Vater adenocarcinomas [21]. To the best of our knowledge, there are however no studies reporting on whether the clinical impact of MMR immunophenotype in periampullary cancer differs by morphological type. I-type tumors bear a resemblance to colorectal cancer, and the results from the present study demonstrate that dMMR is more common in I-type tumors than in PB-type tumors, and confers a prognostic value only in the former, although this did not remain significant in the adjusted model. dMMR was significantly associated with more favorable clinicopathological factors in both I-type and PB-type tumors. Colorectal cancers with dMMR and MSI are known to have a more dense infiltration of intraepithelial activated CD8^+^ tumor-infiltrating lymphocytes than microsatellite stable colorectal cancers [34]. Our results also demonstrate an association between dMMR and CD8^+^ tumor-infiltrating lymphocytes in I-type tumors, and an association between dMMR and CD56^+^ NK/NKT cells in PB-type tumors. A high density of CD56^+^ NK/NKT cells has previously been shown to be significantly associated with a prolonged survival in this cohort [28]. Moreover, and notably, there was a negative interaction between high density of CD56^+^ NK/NKT cells and adjuvant treatment in patients with PB-type tumors, which is similar to the findings related to dMMR status in this study. This observation should however be interpreted with caution, given the small number of cases with dMMR in PB-type tumors, but is still noteworthy and merits validation, since it may be of clinical relevance. This observation is also supported by findings in colorectal cancer, where studies have shown that patients with MSI high tumors have no survival benefit from adjuvant 5-FU-based chemotherapy, and have an even poorer response to 5-FU, compared to patients with pMMR or microsatellite stable tumors [35, 36]. One theory explaining the resistance to 5-FU in dMMR tumors is the overexpression of tumoral thymidylate synthase (TS), which is the main 5-FU target, and dihydropyrimidine dehydrogenase (DPD), which is the key enzyme of 5-FU catabolism [37]. MMR immunophenotype may indeed confer a predictive value in periampullary adenocarcinoma as Riazy et al. recently demonstrated a prolonged disease-specific survival with gemcitabine or 5-FU treatment in patients with pMMR pancreatic cancer (n = 224), but no statistically significant survival advantage for treated patients with dMMR tumors (n = 41) [18]. In the present study it is noteworthy that none of the patients with dMMR PB-type tumors received 5-FU, which might imply that dMMR also signifies resistance to other types of chemotherapeutic agents. A comparative strength of the herein investigated retrospective patient cohort is that it encompasses a large proportion of patients who did not receive any adjuvant chemotherapy, which is due to the fact that all types of periampullary adenocarcinomas are included and that the cohort stretches from 2001 to 2011, thus spanning a period of time where adjuvant treatment was yet not standard of care. Moreover, the proportion of patients with PB-type tumors in this cohort is comparatively large, with 110 patients, whereof almost 50% did not receive adjuvant chemotherapy. Notably, there was no significant difference in the distribution of conventional clinicopathological factors between treated and untreated patients, except for a significantly higher age in the latter. Therefore, although treatment predictive effects are best studied in a randomized setting, the nearly equal distribution of treated and untreated patients in this retrospective cohort provides a good setting for analysis of potential treatment predictive markers, despite the retrospective design. A limitation to this cohort is however that performance status (PS), which is an important clinicopathological factor that might have affected weather patients have received adjuvant treatment or not, has not been registered. However, the finding that patients with dMMR tumors who received adjuvant treatment seemed to do worse than those not receiving adjuvant treatment, implies that PS may not have affected the results. Another limitation is related to the use of TMA, which may not reflect the heterogeneity of the tumor, however this is a well-validated method for studies of biomarkers [38]. It should also be pointed out that a single whole tissue section will also merely represent a part of the tumor, and in the herein analyzed TMA, tissue cores were sampled from different archival blocks with primary tumor, and, when present, different lymph node metastases. Therefore, uniform loss of an MMR protein across multiple TMA-samples should provide similarly reliable evidence of dMMR as analysis of a single whole tissue section. Another issue that needs to be addressed in light of the small sample size is the risk of overfitting the multivariable model. We have however chosen to adjust for known prognostic parameters that were also found to be significant in the univariable analysis.

## Conclusions

The results from this study demonstrate that dMMR is more frequent in I-type compared to PB-type periampullary adenocarcinoma, and is a prognostic factor for long-term survival only in the former. However, despite the small numbers, the finding of dMMR being a potential negative predictor of response to adjuvant chemotherapy in PB-type tumors is noteworthy and merits further validation in larger patient cohorts, as it may be highly relevant for clinical decision-making.

## Additional file

**Additional file 1.** Unadjusted and adjusted hazard ratios of the impact of MMR immunophenotype on recurrence free survival in I-type tumors, PB-type tumors, and the entire cohort.

## Abbreviations

MMR: mismatch repair
dMMR: deficient MMR
pMMR: preserved MMR
PB-type: pancreatobiliary type adenocarcinoma
I-type: intestinal type adenocarcinoma
TMA: tissue microarray
OS: overall survival
RFS: recurrence free survival
HR: hazard ratio
CI: confidence interval
Q1: quartile 1
Q3: quartile 3
