# Supplementary material for: Relationship between mismatch repair immunophenotype and long-term survival in patients with resected periampullary adenocarcinoma
Source: J Transl Med. 2018 Mar 14;16:66. doi: 10.1186/s12967-018-1444-4 (PMC5853113; doi:10.1186/s12967-018-1444-4)
Supplement: Supplementary file 1 — Additional file 1. Unadjusted and adjusted hazard ratios of the impact of MMR immunophenotype on recurrence free survival in I-type tumors, PB-type tumors, and the entire cohort. [file 12967_2018_1444_MOESM1_ESM.docx]

Additional file 1. Unadjusted and adjusted hazard ratios of the impact of MMR immunophenotype on recurrence free survival in I-type tumors, PB-type tumors, and the entire cohort

|  | Intestinal type | | | Pancreatobiliary type | | | All | | |
| --- | --- | --- | --- | --- | --- | --- | --- | --- | --- |
|  |  | Unadjusted | Adjusted |  | Unadjusted | Adjusted |  | Unadjusted | Adjusted |
|  | n(events) | HR(95%CI) | HR(95%CI) | n(events) | HR(95%CI) | HR(95%CI) | n(events) | HR(95%CI) | HR(95%CI) |
| Age |  |  |  |  |  |  |  |  |  |
| Continuous | 63 (31) | 1.00 (0.96-1.04) | - | 109 (93) | 0.98 (0.96-1.01) | - | 172 (124) | 1.02 (0.98-1.02) | - |
| Gender |  |  |  |  |  |  |  |  |  |
| Female | 35 (12)  34 (12)* | 1.00 | 1.00 | 51 (44) | 1.00 | - | 86 (56) | 1.00 | - |
| Male | 28 (19)  27 (18)* | *2.22 (1.07-4.62)* | 1.92 (0.88-4.18) | 58 (49) | 1.10 (0.73-1.66) | - | 86 (68) | 1.43 (0.997-2.04) | - |
| Tumor origin |  |  |  |  |  |  |  |  |  |
| Intestinal | - | - | - |  | - | - | 63 (31)  61 (30)* | 1.00 | 1.00 |
| Pancreatobiliary type | - | - | - |  | - | - | 109 (93)  108 (93)* | *2.68 (1.77-4.04)* | 1.39 (0.87-2.23) |
| Tumor size |  |  |  |  |  |  |  |  |  |
| Continuous | 63 (31) | 0.99 (0.91-6.23) | - | 109 (93)  108 (93) | *1.04 (1.02-1.05)* | 1.02 (0.99-1.04) | 172 (124)  169 (123)* | *1.02 (1.00-1.03)* | 1.00 (0.98-1.02) |
| T-stage |  |  |  |  |  |  |  |  |  |
| T1-T2 | 16 (5) | 1.00 | - | 15 (9)  14 (9)* | 1.00 | 1.00 | 31 (14)  28 (13)* | 1.00 | 1.00 |
| T3-T4 | 47 (26) | 2.39 (0.91-6.23) | - | 94 (84)  94 (84)* | *2.87 (1.44-5.75)* | 1.33 (0.62-2.84) | 141 (110)  141 (110)* | *2.90 (1.66-5.07)* | 1.56 (0.82-2.96) |
| N-stage |  |  |  |  |  |  |  |  |  |
| N0 | 35 (13)  33 (12)* | 1.00 | 1.00 | 32 (24)  31 (24)* | 1.00 | 1.00 | 67 (37)  64 (36)* | 1.00 | 1.00 |
| N1-2 | 28 (18)  28 (18)* | *2.17 (1.06-4.45)* | 0.91 (0.38-2.17) | 77 (69)  77 (69)* | *2.31 (1.44-3.71)* | *1.85 (1.08-3.20)* | 105 (87)  105 (87)* | *2.55 (1.72-3.76)* | 1.52 (0.98-2.36) |
| Differentiation grade |  |  |  |  |  |  |  |  |  |
| Well-moderate | 31 (12) | 1.00 | - | 42 (32)  41 (32)* | 1.00 | 1.00 | 73 (44)  71 (44)* | 1.00 | 1.00 |
| Poor | 32 (19) | 2.01 (0.98-4.15) | - | 67 (61)  67 (61)* | *2.44 (1.56-3.83)* | *2.10 (1.28-3.44)* | 99 (80)  98 (79)* | *2.19 (1.51-3.18)* | *1.63 (1.08-2.46)* |
| Involved margins |  |  |  |  |  |  |  |  |  |
| R0 | 18 (3)  17 (3)* | 1.00 | 1.00 | 7 (5) | 1.00 | - | 25 (8)  23 (8)* | 1.00 | 1.00 |
| R1 & Rx | 45 (28)  44 (27)* | *5.19 (1.57-17.13)* | *3.76 (1.04-13.58)* | 102 (88) | 2.26 (0.92-5.60) | - | 147 (116)  146 (115)* | *4.34 (2.11-8.92)* | *2.30 (1.09-4.84)* |
| Lymphatic growth |  |  |  |  |  |  |  |  |  |
| Absent | 28 (6)  28 (6)* | 1.00 | 1.00 | 35 (26)  34 (26)* | 1.00 | 1.00 | 63 (32)  62 (32)* | 1.00 | 1.00 |
| Present | 35 (25)  33 (24)* | *5.52 (2.25-13.56)* | 2.47 (0.93-6.62) | 74 (67)  74 (67)* | *1.80 (1.14-2.85)* | 1.04 (0.62-1.73) | 109 (92)  107 (91)* | *2.70 (1.80-4.06)* | 1.28 (0.81-2.01) |
| Vascular growth |  |  |  |  |  |  |  |  |  |
| Absent | 58 (26)  56 (25)* | 1.00 | 1.00 | 73 (60)  72 (60)* | 1.00 | 1.00 | 131 (86)  128 (85)* | 1.00 | 1.00 |
| Present | 5 (5)  5 (5)* | *8.32 (2.91-23.78)* | *3.56 (1.17-10.86)* | 36 (33)  36 (33)* | *2.39 (1.53-3.73)* | *1.99 (1.23-3.21)* | 41 (38)  41 (38)* | *3.50 (2.33-5.24)* | *2.17 (1.40-3.36)* |
| Perineural growth |  |  |  |  |  |  |  |  |  |
| Absent | 43 (16)  42 (16)* | 1.00 | 1.00 | 25 (17)  24 (17)* | 1.00 | 1.00 | 68 (33)  66 (33)* | 1.00 | 1.00 |
| Present | 20 (15)  19 (14)* | *2.71 (1.33-5.51)* | 0.46 (0.14-1.50) | 84 (76)  84 (76)* | *2.48 (1.45-4.25)* | 1.31 (0.72-2.37) | 104 (91)  103 (90)* | *3.26 (2.17-4.91)* | 1.10 (0.68-1.76) |
| Growth in peripancreatic fat |  |  |  |  |  |  |  |  |  |
| Absent | 42 (14)  40 (13)* | 1.00 | 1.00 | 25 (17)  24 (17)* | 1.00 | 1.00 | 67 (31)  64 (30)* | 1.00 | 1.00 |
| Present | 21 (17)  21 (17)* | *4.20 (2.04-8.65)* | *4.90 (1.47-16.31)* | 84 (76)  84 (76)* | *2.24 (1.31-3.81)* | 1.27 (0.70-2.29) | 105 (93)  105 (93)* | *3.66 (2.41-5.56)* | 1.58 (0.95-2.63) |
| Adjuvant treatment |  |  |  |  |  |  |  |  |  |
| None | 45 (23) | 1.00 | - | 50 (41) | 1.00 | - | 95 (64) | 1.00 | - |
| Any | 18 (8) | 0.77 (0.34-0.71) | - | 59 (52) | 1.09 (0.72-1.64) | - | 77 (60) | 1.22 (0.85-1.73) | - |
| MMR |  |  |  |  |  |  |  |  |  |
| pMMR | 49 (29)  49 (29)* | 1.00 | 1.00 | 101 (88)  101 (88)* | 1.00 | 1.00 | 149 (116)  149 (116)* | 1.00 | 1.00 |
| dMMR | 12 (1)  12 (1)* | *0.09 (0.01-0.69)* | 0.19 (0.02-1.50) | 7 (5)  7 (5)* | 0.53 (0.23-1.22) | 1.14 (0.42-3.06) | 20 (7)  20 (7)* | *0.26 (0.12-0.55)* | 0.46 (0.19-1.13) |

Italics emphasis indicate a p-value <0.05.

*Number and events for the adjusted analysis
